# Function and clinical significance of N6-methyladenosine in digestive system tumours

**DOI:** 10.1186/s40164-021-00234-1

**Published:** 2021-07-10

**Authors:** Junchao Huang, Yingjie Shao, Wendong Gu

**Affiliations:** Department of Radiation Oncology, The Third Affiliated Hospital of Soochow University, 185 Juqian Street, Changzhou, 213003 China

## Abstract

RNA modification, like DNA methylation, histone modification, non-coding RNA modification and chromatin rearrangement, plays an important role in tumours. N6-methyladenosine (m6A) is the most abundant RNA modification in cells, and it regulates RNA transcription, processing, splicing, degradation, and translation. m6A-associated proteins have been used as new biomarkers and therapeutic targets for tumour prediction and monitoring. There are three main types of proteins involved in m6A methylation: methyltransferases (METTL3, METTL14, WTAP, RBM15, ZC3H13 and KIAA1429), demethylases (FTO, ALKBH5 and ALKBH3) and RNA-binding proteins (YTHDF1-3, YTHDC1-2, IGF2BPs and HNRNPs). This article reviews the origins, characteristics and functions of m6A and its relationship with digestive system tumours based on recent research. The expression of m6A regulators can be used as an evaluation indicator of tumour growth and progression and as a prognostic indicator. In-depth research on m6A methylation in digestive system tumours may provide new directions for clinical prediction and further treatment.

## Introduction

Posttranscriptional regulation is ubiquitous in cells, and RNA methylation is a widespread type of epigenetic modification along with DNA methylation, histone modification, non-coding RNA modification and chromatin rearrangement [1]. Although N6-methyladenosine (m6A) was first discovered in the 1970s [2], the development of related research was restricted due to the lack of available methods for mapping its precise transcript location in transcripts and the lack of knowledge about cytokines related to the regulation of its production and modification. It was not until the discovery of the genome-wide m6A mapping method that m6A began to attract the attention of researchers. Since then, m6A has been widely studied as a broad regulatory mechanism that can dynamically and reversibly regulate various physiological processes.

**Fig. 1:**
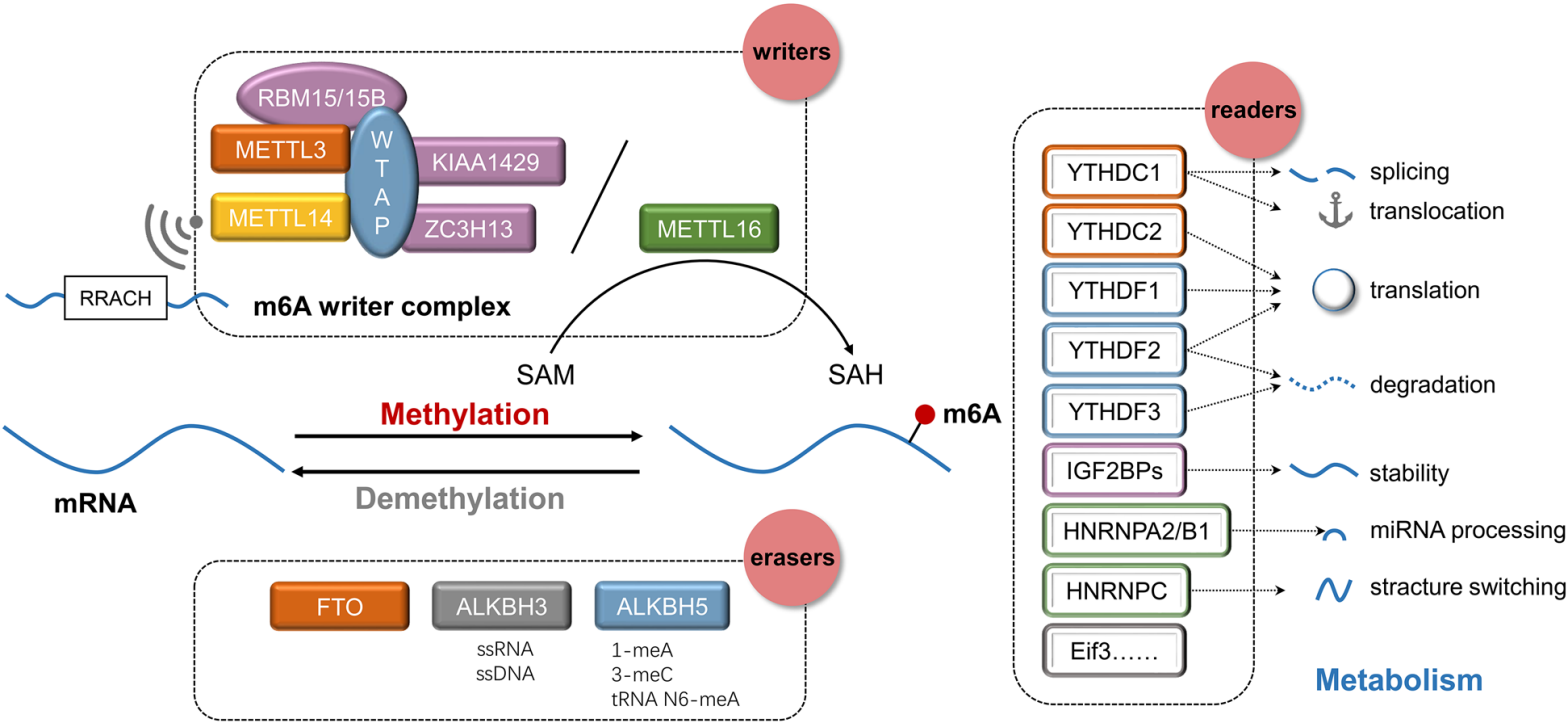
The molecular mechanism of m6A in cancer

There are three main types of proteins involved in m6A methylation: methyltransferases, demethylases and RNA-binding proteins (Fig. 1). Methyltransferases are also called “writers” and form stable complexes to catalyze m6A methylation of bases in mRNAs, mainly through interactions involving methyltransferase-like protein 3 (METTL3) [3, 4], methyltransferase-like protein 14 (METTL14) [5], Wilms’ tumour 1-associated protein (WTAP) [6], RNA-binding motif protein 15 (RBM15), RNA-binding motif protein 15B (RBM15B) [7], virlike m6A methyltransferase associated (VIRMA/KIAA1429) [8], zinc finger CCCH-Type containing 13 (ZC3H13) [9], methyltransferase-like protein 16 (METTL16) [10] and other core proteins. METTL14 and METTL3 form a stable heterodimer core complex that can catalyze the transfer of meth1 groups [5, 11]. METTL3 is the most important component of the m6A methyltransferase complex (MTC) and is highly conserved in eukaryotes from yeast to humans [3]. The main role of METTL14 is to stabilize the structure of the MTC and recognize specific RRACH motifs as a catalytic substrate [11]. More interestingly, METTL3 is both a writer and a reader, and it can directly enhance mRNA translation [4]. WTAP has no catalytic activity but acts as a regulatory subunit in the m6A methyltransferase complex. m6A modification is mainly promoted through the recruiting METTL3 and METTL14 into the nucleus [6]. RBM15 and RBM15B have no catalytic function but can bind to METTL3 and WTAP to direct these two proteins to specific RNA sites for m6A modification [7, 12]. KIAA1429 mediates m6A methylation of mRNAs near the 3′-UTR and stop codon. KIAA1429 can recruit the core components of the methyltransferase complex and interact with the polyadenylation cleavage factors CPSF5 and CPSF6 [13]. Except for METTL3, all components of MTC lack RNA methyltransferase activity. The sites that rely on METTL16 are mainly located in introns or intron-exon boundaries, unlike the common m6A site in UTRs. METTL16 regulates the expression of human MAT2A, which encodes S-adenosylmethionine (SAM) synthase and is expressed in most cells [10]. SAM is an important metabolite and a methyl donor for DNA and histone methylation that is able to control the regulation of gene expression [14].

Demethylases, known as “erasers”, ensure that m6A methylation is a dynamic and reversible process. Fat mass and obesity-associated protein (FTO) [15], ALKBH3 and ALKBH5 [16] can selectively remove m6A marks from target mRNA. FTO was the first demethylase discovered in 2011. He et al. found that siRNA-mediated knockdown of FTO expression enhanced the mRNA m6A level while the upregulation of FTO gene expression inhibits m6A methylation, thus revealing the demethylase activity of FTO [15]. The discovery of FTO revealed that m6A methylation is a reversible process. ALKBH5, another mammalian demethylase, can oxidatively reverse m6A methylation of mRNA in vitro and in vivo. The demethylase activity of ALKBH5 significantly affects mRNA output, RNA metabolism, and the assembly of mRNA processing factors in nuclear speckles [16]. ALKBH5 has a catalytic domain able to demethylate single-stranded RNA (ssRNA) and single-stranded DNA (ssDNA), in particular, to catalyse the demethylation of m6A in ssRNA, supporting that the methylation of m6A is reversible in RNA [17]. ALKBH3 can demethylate 1-meA and 3-meC in RNA and N6-meA in tRNA, and ALKBH3-modified tRNA can increase protein translation efficiency [18].

RNA-binding proteins, known as “readers,” can decode m6A marks and generate functional signals [19], and these proteins include YT521-B homology domain family proteins 1, 2, and 3 (YTHDF1, YTHDF2, YTHDF3, respectively); YT521-B homology domain containing 1 and 2 (YTHDC1 and YTHDC2, respectively) [20]; eukaryotic translation initiation factor 3 H (Eif3) [21]; insulin-like growth factor 2 mRNA-binding proteins (IGF2BPs, including IGF2BP1/2/3) [22]; and heterogeneous nuclear ribonucleoproteins (HNRNPs, including HNRNPA2/B1, HNRNPC/G) [23]. YTHDF2 was the first m6A reader discovered to recognize a specific m6A site through its C-terminal region, and its N-terminal region binds to the SH domain of CCR4-NOT transcription complex subunit 1 (CNOT1), thereby directly recruiting the CCR4-NOT deadenylation complex. Finally, the m6A-modified RNA is transferred to the processing body, thus facilitating its degradation [24]. In contrast to YTHDF2, YTHDF1 can promote the translation of m6A-modified mRNA [25].

Previously, some articles have discussed the relevant literature on the role of m6A in digestive system tumours [26–29], but as the literature on m6A has been rapidly updated in recent years, it is necessary to review it again. Therefore, the latest research progress and clinical applications of m6A in digestive system tumours are reviewed in this paper. Table 1 shows the m6A-associated proteins related to digestive system tumours and their possible functions.

**Table 1:** The roles of m6A-associated proteins in digestive system tumours

Tumour type	m6A Regulators	Role in M6A	Mechanism	Function in cancer	Reference
ESCC	FTO	Eraser	Targets MMP13	Promotes tumour progression	[32]
	METTL3	Reader	Activates Wnt3/β-catenin and AKT signaling pathway	Promotes tumour proliferation and invasion	[33]
	ALKBH5	Eraser	–	Inhibits tumour progression	[36]
	HNRNPC	Reader	–	Promotes tumour progression	[36]
	HNRNPA2B1	Reader	Up-regulates fatty acid synthase ACLY and ACC1	Promotes tumour proliferation, metastasis and invasion	[35]
	YTHDC2	Reader	–	–	[37]
GC	METTL3	Writer	Promotes the m6A modification of GFI1	Promotes tumour cell proliferation and migration	[44]
			METTL3/HDGF/GLUT4/ENO2 pathway	Promotes tumour progression	[46]
			METTL3/ZMYM1/E-cadherin pathway	Promotes tumour progression	[45]
			METTL3/BATF2/p53/ERK pathway	Promotes tumour proliferation and metastasis	[48]
			Promotes the m6A modification of MYC	Promotes tumour progression	[51]
			Regulates several components of MYC gene (such as MCM5, MCM6, etc.)	–	[50]
	METTL14	Writer	PI3K/AKT/mTOR pathway	Inhibits tumour proliferation and invasion	[53]
	YTHDF1	Reader	YTHDF1/FZD7/β-catenin pathway	Promotes tumour progression	[54]
	WTAP	Writer	–	Promotes tumour progression	[60]
	FTO	Eraser	–	Promotes tumour proliferation, metastasis and invasion	[55]
	ALKBH5	Eraser	Decreases methylation of the lncRNA NEAT1	Promotes tumour metastasis and invasion	[143]
CRC	METTL3	Writer	METTL3/IGF2BP2/SOX2 pathway	Promotes tumour progression	[63]
			Stabilizes CCNE1 mRNA	Promotes tumour progression	[64]
			METTL3/miR-1246/SPRED2 /MAPK pathway	Promotes tumour progression	[65]
			Suppresses SOCS2 expression	Promotes tumour progression	[66]
			METTL3/HK2/GLUT1/IGF2BPs pathway	Promotes tumour progression	[67]
			METTL3/GLUT1/mTORC1 pathway	Promotes tumour progression	[144]
			P38 / ERK pathway	Inhibits tumour progression	[62]
			The expression of p53 missense mutation	Acquired multidrug resistance	[69]
	METTL14	Writer	Inhibits the expression of SOX4	Inhibits tumour progression	[70]
			METTL14/miR-375/YAP1 pathway	Inhibits tumour metastasis	[71]
			METTL14/miR-375/SP1 pathway	Inhibits tumour metastasis and invasion	[71]
			METTL14/YTHDF2/lncRNA pathway	Inhibits tumour progression	[72]
	YTHDF1	Reader	Up-regulates the expression of FZD9 and WNT6 and activate Wnt/β-catenin pathway	Promotes tumour progression	[81]
	IGF2BP2	Reader	RNA-protein ternary complex	Promotes tumour progression	[79]
	IGF2BP1	Reader	Upregulates the stability and expression of c-Myc mRNA	Promotes tumour progression	[78]
	FTO	Eraser	Removes the m6A modification of MYC and enhance MYC expression	Promotes tumour proliferation, metastasis and invasion	[76]
HCC	WTAP	Writer	HuR/ETS1/p21/p27 pathway	Promotes tumour progression	[88]
	KIAA1429	Writer	Inhibits ID2 expression	Promotes tumour metastasis and invasion	[89]
			Degrade GATA3 pre-mRNA	Promotes tumour proliferation and metastasis	[90]
			KIAA1429/circ-KIAA1429/ZEB1 pathway	Promotes tumour proliferation, metastasis and invasion	[91]
	YTHDF1	Reader	PI3K/AKT/mTOR pathway	Promotes tumour proliferation	[93]
	YTHDF3	Reader	M6A/YTHDF3/ZEB1 pathway	Promotes tumour proliferation, metastasis and invasion	[91]
	YTHDF2	Reader	Degrades IL11 and SERPINE2 mRNA	Inhibits tumour proliferation	[94]
			Degrades EGFR mRNA	Inhibits tumour progression	[95]
			–	Promotes tumour metastasis	[96]
	METTL14	Writer	Up-regulates miR-126 expression	Inhibits tumour metastasis	[97]
	METTL3	Writer	UBC9/SUMOylated METTL3/Snail pathway	Promotes tumour progression	[106]
			METTL3/miR-873-5p/SMG1 pathway	Promotes tumour progression	[107]
			METTL3/IGF2BP2/FEN1 pathway	Promotes tumour progression	[102]
			METTL3/HBXIP/HIF-1α pathway	Promotes tumour progression	[105]
			METTL3/YTHDF2/SOCS2 pathway	Promotes tumour progression	[100]
			Upregulated LINC00958	Promoted HCC lipogenesis and progression	[112]
	ZCCHC4	Writer	–	Promotes tumour proliferation	[103]
	ALKBH5	Eraser	ALKBH5 / LYPD1 pathway	Inhibits tumour proliferation and invasion	[104]
	FTO	Eraser	–	Inhibits tumour progression	[111]
PDAC	METTL3	Writer	–	Promotes tumour proliferation and invasion	[122]
			METTL3/miR-25-3p/PHLPP2-AKT pathway	Promotes tumour progression	[124]
	METTL14	Writer	Decreases PERP mRNA expression	Promotes tumour proliferation and migration	[123]
	YTHDF2	Reader	–	Promotes tumour proliferation	[125]
	ALKBH5	Eraser	Post-transcriptionally activates PER1	Inhibits tumour proliferation, metastasis and invasion	[126]
			Demethylates lncRNA KCNK15-AS1	Inhibits tumour metastasis	[127]
			Down-regulates the m6A level of WIF-1 and inhibit the activation of Wnt pathway	Inhibits tumour progression	[128]
	FTO	Eraser	Stabilizes bHLH mRNA	Promotes tumour proliferation	[129]
	IGF2BP2	Reader	Up-regulates lncRNA DANCR expression	Promotes tumour proliferation	[130]

*ESCC* esophageal squamous cell cancer, *GC* gastric cancer, *CRC* colorectal cancer, *HCC* hepatocellular carcinoma, *PDAC* pancreatic ductal adenocarcinoma

### The role of m6A in digestive system tumours

#### Esophageal cancer (EC)

EC is the seventh leading cause of cancer (604,000 new cases per year) and the sixth leading cause of cancer deaths (544,000 deaths per year) [30]. Histologically, EC is mainly classified into squamous cell carcinoma (SCC) and adenocarcinoma (ADCA). In the last 3 decades, the incidence of SCC has decreased to less than 30 % in the United States, while the incidence of ADCA has increased to above 60 %. 90 % of esophageal squamous cell carcinoma (ESCC) cases in the United States are caused by smoking, alcohol abuse, or insufficient intake of fruits and vegetables. Most esophageal ADCAs originate from Barrett’s metaplastic tissue, and 80 % of these tumours are attributed to a history of smoking, a high body mass index, gastroesophageal reflux disease (GERD), and an insufficient intake of fruits and vegetables. Alcohol consumption is not correlated with ADCA [31]. Although m6A modification is closely related to the occurrence and development of cancer, the relationship between EC and m6A modification was revealed only recently. In 2019, Liu et al. confirmed for the first time that FTO plays a role in the occurrence and development of ESCC, and they demonstrated through qRT-PCR and Western blotting that MMP13 might act as a downstream effector of FTO in ESCC [32]. Subsequent studies also found that knocking out METTL3 can inhibit the invasive progression of human EC in vitro, and researchers inferred that this inhibition might be mediated by blockade of the Wnt3/β-catenin and AKT signalling pathways [33]. Xia et al. also confirmed that, unlike the low METTL3 phenotype, the high METTL3 phenotype is significantly positively correlated with the poor prognosis of ESCC, and they proposed that METTL3 expression level is an independent predictor of disease-free survival (DFS) and overall survival (OS) in patients with ESCC [34]. HNRNPA2B1 is an oncoprotein carcinogen that promotes the progression of ESCC by upregulating the expression of the fatty acid synthases ACLY and ACC1. HNRNPA2B1 knockout can inhibit the proliferation, migration and invasion capacities of ESCC cells. In addition, the expression of HNRNPA2B1 is positively correlated with the tumour diameter and lymphatic metastasis in ESCC [35]. ALKBH5 and HNRNPC have also been confirmed to be related to the prognosis of EC [36]. YTHDC2 was found to have predictive value for the risk of ESCC [37]. It has also been suggested that m6A modification of LINC00278 regulates the translation of YY1BM, which in part leads to the higher incidence of ESCC in men than in women [38]. Most of the research on m6A modification in EC has focused on ESCC, and studies focusing on ADCA are rare. We eagerly anticipate more research on ADCA and the identification of related target genes for the treatment of EC.

#### Gastric cancer (GC)

MiRNAs are a group of conserved non-coding endogenous RNA transcripts complementary to 3′ untranslated regions (3′-UTRs), and miRNAs can trigger mRNA degradation or translation inhibition, thus preventing their target genes from being translated into functional proteins [39]. Various miRNAs have prognostic significance and regulatory functions in GC. It has recently been found that m6A modifications are widely present in miRNAs, affecting the prognosis of tumour development. MiR-660 regulates cell proliferation in GC by regulating the oncogene E2F3. However, there is an m6A-modified sequence in the area near the binding site of miR-660 and E2F3, and if the m6A-modified sequence is mutated, miR-660 and E2F3 cannot bind. m6A modification is essential for the inhibitory effect of miR-660 on E2F3 [40]. It has been found that miR-4429 can reduce the m6A-induced stability of Sec. 62 by targeting the METTL3 binding site, thereby preventing the progression of GC and providing innovative ideas for the targeted molecular therapy of GC [41]. m6A can also promote the processing of pri-miR-17-92 into the miR-17-92 cluster through an m6A/DGCR8-dependent mechanism. The miR-17-92 cluster can activate the AKT/mTOR pathway by targeting PTEN or TMEM127. The mTOR inhibitor everolimus has a better effect on high-METTL3 GC than on low-METTL3 GC [42]. A consensus has been reached that METTL3 is an oncogene in GC [43–46]. Downregulation of METTL3 expression can reduce the metastasis and invasion abilities of GC cells [44]. Zhang et al. found that changes in m6A modification may promote the progression of GC [47]. In addition, METTL3 can promote glycolysis and angiogenesis through the METTL3/HDGF/GLUT4/ENO2 axis, thus promoting the occurrence and development of GC [46]. Some scholars have found that METTL3 produces cancer-promoting effects through the METTL3/ZMYM1/E-cadherin axis in GC [45]. Downregulation of METTL3 can inhibit the growth and metastasis of GC by regulating cell cycle progression and ECM degradation through the METTL3/BATF2/p53/ERK axis. Moreover, BATF2 plays a tumour suppressor role in the development of GC [48]. MYC is an oncogene involved in cell cycle regulation, cell growth arrest, cell adhesion, metabolism, ribosome biogenesis, protein synthesis and mitochondrial function. MYC has been described as a key element in several carcinogenic processes in humans. There is an association between MYC dysregulation and GC [49]. Several MYC target genes (such as MCM5 and MCM6) in GC are regulated by METTL3 [50]. HBXIP can also play an oncogenic role in GC through the MYC mRNA m6A modification mediated by METTL3 [51]. The abovementioned studies demonstrate that METTL3 exerts oncogenic effects through various different pathways. It is expected that an increasing number of downstream genes will be discovered in the future, thus identifying antagonistic genes of METTL3. FTO can participate in the regulation of GC by HDAC3 through the FTO/m6A/MYC axis, and HDAC3 can promote the growth, migration and invasion of GC cells by degrading FOXA2 [52]. METTL14 overexpression inhibits the proliferation and invasion capabilities of GC cells by inactivating the PI3K/AKT/mTOR pathway and the epithelial-mesenchymal transition (EMT) pathway, respectively [53]. Pi et al. directly demonstrated for the first time that m6A mRNA methylation can regulate the Wnt/β-catenin pathway and control the progression of GC. The m6A-dependent YTHDF1-FZD7-β/catenin axis plays a role in promoting the development of GC. Pi et al. proposed that this finding may also be applicable to other cancers with enhanced Wnt/β-catenin activity [54]. Most of the research on other m6A regulators has focused on prediction and prognostic evaluation. For example, according to tissue microarray-immunohistochemistry (TMA-IHC) staining in GC tissues, abnormally high mRNA expression of the demethylase genes FTO and ALKBH1 mRNA was related to poor OS, while the protein expression of FTO and ALKBH1 was significantly downregulated. In addition, lower ALKBH1 protein expression levels were closely related to tumour size (≥ 5 cm) and high TNM stage (III/IV). Decreased FTO protein expression is associated with short OS in patients with GC [55]. Guan et al. analysed The Cancer Genome Atlas (TCGA) data using the Kaplan-Meier method and found that the upregulation of WTAP and FTO expression is significantly related to the poor prognosis of patients with GC [56]. Scholars have established a prediction model based on the expression of FTO and RBM15. In this model, patients with GC were divided into “high-risk” and “low-risk” groups to compare differences in survival. The survival time in the high-risk group was significantly decreased [57]. Ge et al. also innovatively proposed the detection of m6A levels in peripheral blood RNA as a potential reliable biomarker for GC diagnosis and follow-up and found that an increased peripheral blood m6A levels in patients with GC was accompanied by downregulation of the demethylases ALKBH5 and FTO [58]. YTHDF1 is mutated in approximately 7 % of patients with GC, and high expression of YTHDF1 is associated with more aggressive tumour progression and poorer overall survival [54]. YTHDF1 is significantly related to the high-risk GC subtypes, and YTHDF1 may be a potential target for the early diagnosis of GC [59]. However, further research on the mechanism of these regulatory genes is needed. High WTAP expression is correlated with a poor survival prognosis. Weighted correlation network analysis and enrichment analysis further confirmed that high WTAP expression is related to RNA methylation, and low WTAP expression is often related to higher T cell-related immune responses [60]. Bo Zhang et al. proposed that m6A modification also plays an important role in the diversity and complexity of the tumour microenvironment and identified three different m6A modification modes. The infiltration characteristics of tumour microenvironment (TME) infiltration characteristics in the three modes are highly consistent with the three immunophenotypes of tumour immune rejection, immune inflammation and immune desert. Evaluation of m6A modification patterns in a single tumour was found to predict the stage, subtype, TME mesenchymal activity, genetic variation and patient prognosis in the presence of tumour inflammation. A low m6A score is characterized by increased mutation load and increased immune activation, suggesting that the TME has an inflammatory phenotype, and the 5-year survival rate is 69.4 %. Matrix activation and a lack of effective immune infiltration were observed in the high m6A score subtype, indicating that this subtype has a non-inflammatory and immune-rejection TME phenotype with a low survival rate. A low m6A score is also related to an increased neoantigen load and an enhanced response to anti-PD-1/L1 immunotherapy. Analysis of two immunotherapy cohorts confirmed that patients with a lower m6A score exhibited significant therapeutic advantages and clinical benefits [61]. This research may be able to guide the clinical use of immunotherapeutic drugs in the future. Overall, the progress of research on the role of m6A in GC is satisfying, and many indicators that can be used for clinical prognostic evaluations have been identified. It is expected that related clinical trials will be carried out in the future to accelerate the clinical translation of this knowledge.

#### Colorectal cancer (CRC)

METTL3 is also an important player in CRC. Ruet al. showed that METTL3 acts as a tumour suppressor gene in CRC and may affect the progression of CRC through the p38/ERK signalling pathway [62]. However, Li et al. found the opposite results. They suggested that METTL3 plays a role in promoting CRC development. METTL3 acts on the reader IGF2BP2, and IGF2BP2 directly binds to a specific m6A site of in SOX2 CDS and controls the half-life of SOX2 mRNA by relying on m6A modification to produce cancer-promoting effects [63]. Scholars later confirmed results of the study by Li et al. and further revealed that METTL3 could directly stabilize CCNE1 mRNA in an m6A-dependent manner, thus promoting the proliferation of CRC cells [64]. Peng et al. found that the upregulation of METTL3 expression causes abnormal m6A modification in CRC. The METTL3/miR-1246/SPRED2 axis plays an important role in tumour metastasis as abnormal m6A modification of in CRC leads to the upregulation of METTL3 expression, and pri-miR-1246 can be further processed. This increases the expression of miRNA-1246, associated with metastasis, resulting in the downregulation of the expression of the anti-oncogene SPRED2 and leading to tumour metastasis [65]. The increase in METTL3 levels may maintain the tumorigenicity of colon cancer cells by inhibiting SOCS2 [66]. In addition, m6A modification is closely related to the activation of the glycolytic pathway in the tissues of patients with CRC. METTL3 stabilizes the expression of HK2 and SLC2A1 (GLUT1) in CRC through a mechanism dependent on the m6A-IGF2BP2/3-METTL3/HK2/GLUT1-IGF2BPs axis, which plays a key role in CRC pathogenesis. HK2/GLUT1 is regulated by m6A modification and participates in the activation of glycolysis in CRC. METTL3 and its target genes may be markers that can be used to guide the early diagnosis and treatment of CRC in the future [67]. METTL3 can promote CRC by activating the m6A-GLUT1-mTORC1 axis [68]. Additionally, METTL3-mediated m6A modification at a p53 codon harboring a missense mutation regulated the expression of p53 and resulted in acquired multidrug resistance in colon cancer cells [69]. METTL14 is considered to play a tumour suppressor role in CRC [70, 71]. Chen et al. found that METTL14 epigenetically inhibits SOX4 expression through an m6A-YTHDF2-dependent mechanism. The discovery of the METTL14/SOX4 axis and its impact on CRC metastasis will facilitate future CRC research to explore effective treatment strategies [70]. Furthermore, it has been confirmed that METTL14 inhibits the growth of CRC cells through the miR-375/YAP1 pathway and suppresses the migration and invasion of CRC cells through the miR-375/SP1 pathway [71]. Subsequently, the METTL14-YTHDF2-lncRNA axis was discovered in CRC [72]. The emergence of immunotherapy has recently advanced the field of cancer treatment. However, most patients receiving immune checkpoint blockade (ICB) therapy with anti-PD-1 antibodies do not respond or develop resistance, which is a daunting challenge [68]. Wang et al. showed that the absence of METTL3 or MMETTL3 renders CRC and melanoma cells sensitive to anti-PD-1 treatment. In CRC, this sensitization is mediated by the increase in the expression of Stat1 and Irf1. Transcripts are stabilized by the reduction of m6A enrichment. Inhibition of m6A modification can render tumours sensitive to immunotherapy by changing the tumour microenvironment and recruiting CD8 + TILs. The effect of mettl3/14 deletion on tumour growth inhibition is equivalent to that of multiple combination immunotherapy regimens (anti-PD-1 + anti-CTLA-4). In addition, deletion of METTL3 or METTL14 increases sensitivity to interferon-γ treatment (activation of the IFN-γ pathway, which has been shown to be an important indicator of the PD-1 blockade effect [73]), which may combine immunotherapy with newly developed methyltransferase inhibitors for the treatment of CRC [74]. Ni et al. found that there is a negative functional loop formed by the lncRNA-GAS5-YAP-YTHDF3 axis in CRC. Mechanistically, the lncRNA GAS5 directly binds to YAP to promote its phosphorylation and ubiquitin-mediated degradation, thereby weakening YAP-mediated transcription of YTHDF3. YTHDF3 reversibly and selectively binds to m6A-methylated GAS5 to trigger its decay and form the negative feedback loop [75]. FTO enhances the ability of MYC to stimulate the proliferation and invasion of CRC cells and to inhibit their apoptosis. This effect enhances the expression of MYC by removing the m6A modification from MYC in CRC cells [76]. RP11 (a new lncRNA, namely, RP11-138 J23.1) is induced by m6A and can be post-translationally upregulated by Zeb1 to trigger the spread of CRC [77]. Later, it was found that the lncRNA LINC00266-1 encodes the peptide RBRP, which has a cancer-promoting effect in tumourigenesis. As another regulatory subunit of the m6A reader, the RBRP peptide can enhance m6A recognition of m6A on RNA (such as c-Myc mRNA) through the m6A reader IGF2BP1, thereby increasing the stability and expression of c-Myc mRNA [78]. CircNSUN2 enhances the stability of HMGA2 mRNA and promotes the metastasis of CRC to the liver by forming the circNSUN2/IGF2BP2/HMGA2 RNA-protein ternary complex in the cytoplasm [79]. Nishizawa et al. first reported the important role of YTHDF1 in CRC and found that c-Myc has a cancer-promoting effect on the transactivation of the m6A reader YTHDF1, but the specific mechanism is still unclear [80]. Yang Bai et al. found through TCGA and Gene Expression Omnibus (GEO) online databases that an increase in copy number is the main mechanism leading to the overexpression of YTHDF1 in CRC. YTHDF1 can promote CRC. Inhibiting the expression of YTHDF1 downregulates the expression of FZD9 and WNT6 and inhibits the activity of the Wnt/β-catenin pathway [81]. Changes in the levels of the Wnt pathway components can lead to the occurrence of CRC [82]. Song et al. found that HSF1 expression is upregulated in murine tumours driven by activated WNT/β-catenin signalling and in human CRC tissue. It is believed that β-catenin inhibits the expression of miR-455-3p, which targets HSF1, an effect that is beneficial for binding of METTL3 and m6A modification of HSF1 mRNA, thereby promoting HSF1 translation. Targeting HSF1 is a potential strategy for the management of cancers related to activated Wnt/β-catenin signaling in humans [83]. ANKLE1 was identified as a new CRC susceptibility gene with the ability to maintain genomic stability. Tian et al. demonstrated for the first time that coding mutations could change the level of protein expression by affecting the level of RNA m6A modification, thereby triggering a risk of CRC development. It has been found through integrated analysis of comprehensive m6A-related variants, large-scale population studies and a series of functional experiments that an increased m6A level in ANKLE1 mRNA leads to upregulation of ANKLE1 protein expression and the formation of missense variants that are associated with a decline in the risk of CRC [84]. mRNA m6A modification can regulate gene expression in CRC and an affect tumour progression and the survival of patients with CRC [85]. Abnormal expression of WTAP and FTO is significantly related to the progression of CRC, and YTHDC2 and ALKBH5 have been identified as key regulators that independently predict the prognosis of patients with CRC [86]. High METTL3 expression and downregulation of METTL14, METTL16, FTO and ALKBH5 expression are positively correlated with poor prognosis according to analysis of TCGA data [87].

#### Hepatocellular carcinoma (HCC)

Chen et al. reported for the first time that WTAP acts as an oncogene in HCC [88]. WTAP expression is significantly upregulated in HCC and promotes its development. WTAP-guided m6A modification promotes the development of HCC through the HuR-ETS1-p21/p27 axis. Upregulation of WTAP expression contributes to the m6A modification of ETS1 with subsequent by epigenetic silencing of ETS1 through a Hu-Antigen R (HuR)-related mechanism [88]. KIAA1429 inhibits ID2 by upregulating ID2 mRNA m6A modification and promotes the migration and invasion capacity of HCC [89]. Lan et al. found that KIAA1429 is significantly upregulated in HCC tissues. High expression of KIAA1429 in patients with HCC is associated with poor prognosis through the KIAA1429-GATA3 pathway. Silencing KIAA1429 expression can inhibit the proliferation and metastasis of HCC cells in vivo and in vitro, as GATA3 is the direct downstream target of KIAA1429. KIAA1429 induces m6A methylation in the 3’-UTR of GATA3 pre-mRNA, which leads to the separation of the RNA-binding protein HuR and the degradation of GATA3 pre-mRNA. The lncRNA GATA3-AS, transcribed from the antisense strand of the GATA3 gene, serves as a cis-acting element for the preferential interaction between KIAA1429 and GATA3 pre-mRNA. The growth and metastasis of tumours driven by KIAA1429 or GATA3-as are all mediated by GATA3 [90]. It was later found that the expression of hsa_circ_0084922 (derived from KIAA1429, also known as circ_KIAA1429) is upregulated in HCC cells and tumour tissues. The overexpression of circ_KIAA1429 can promote HCC migration, invasion and EMT. Zeb1 is the downstream target of circ_KIAA1429. Upregulation of Zeb1 expression results in HCC cell metastasis induced by circ_KIAA1429. In addition, Wang et al. found that YTHDF3 enhances the stability of Zeb1 mRNA in an m6A-dependent manner, maintaining Zeb1 expression through the m6A-YTHDF3-Zeb1 axis [91]. YTHDF1 expression is also significantly upregulated in liver cancer, and it is positively correlated with pathological stage [92, 93]. Mechanistically, YTHDF1 promotes the growth of HCC cells by activating the PI3K/AKT/mTOR signaling pathway [93]. YTHDF2, another member of the YTH family, acts as a tumour suppressor gene. Silencing YTHDF2 expression in human HCC cells can cause tumour inflammation, vascular remodeling and metastasis. Mechanistically, YTHDF2 degrades m6A-modified IL-11 and SERPINE2 mRNA, resulting in inflammation-mediated malignant tumours and vascular normalization. Accordingly, the transcription of YTHDF2 is inhibited by HIF-2α. Administration of a HIF-2α antagonist (PT2385) can restore the epigenetic mechanism programmed by YTHDF2 and suppress liver cancer [94]. Zhong et al. found that YTHDF2 can also directly bind to m6A-modified site in the EGFR 3’-UTR, thus promoting the degradation of EGFR mRNA in HCC cells and the inhibition of cancer growth and proliferation. It was also proposed that YTHDF2 plays different roles in tumours due to the degradation of its different target mRNAs [95]. However, Zhang et al. suggested that tumour metastasis is a more reasonable indicator of tumour progression than is tumour growth. They used an orthotopic transplantation model to clarify the role of YTHDF2 in promoting tumour metastasis and found that YTHDF2 expression was negatively correlated with patient survival [96]. In contrast, METTL14 plays a role in inhibiting HCC metastasis. m6A modification is reduced in liver cancer, especially in metastatic liver cancer. MiR126 is the downstream target of METTL14. Mechanistically, METTL14 interacts with the microprocessor protein DGCR8 and positively regulates the processing of primary microRNA 126 in an m6A-dependent manner, thereby inhibiting metastasis [97]. In addition, patients with overexpression of METTL14 showed a better prognosis in HCC based on an analysis of TCGA and GEO datasets [98]. MiR-145 can regulate the m6A levels by targeting the 3’-UTR of YTHDF2 mRNA in HCC cells, thereby exerting a tumour suppressor effect. YTHDF2 recognizes the m6A site in an mRNA and mediates its degradation, resulting in a decrease in the m6A level. MiR-145 regulates the action of the m6A reader protein YTHDF2 by targeting its mRNA 3’-UTR of its mRNA, resulting in increased mRNA methylation in HCC cells [99]. METTL3 is frequently upregulated in human HCC and mediates its progression. METTL3 inhibits the expression of SOCS2 through an m6A-YTHDF2-dependent mechanism, which leads to tumour development [100]. Ma et al. discovered a novel methyltransferase, ZCCHC4, which mainly methylates human 28 S rRNA and modifies A4220 in 28 S rRNA. FEN1 is a multifunctional structure-specific nuclease that plays a key role in maintaining the stability of the human genome [101]. Pu et al. confirmed the carcinogenic effect of FEN1 in liver cancer and indicated that it might act through the METTL3-IGF2BP2-FEN1 axis [102]. The ZCCHC4 protein is overexpressed in HCC tumours, and ZCCHC4 knockout was found to significantly reduce the tumour size in mouse xenograft models [103]. ALKBH5 has been identified as a tumour suppressor in HCC that can inhibit its proliferation and invasion. ALKBH5-mediated m6A demethylation leads to post-transcriptional inhibition of LYPD1, which can be recognized and stabilized by the m6A reader IGF2BP1. Dysregulation of the ALKBH5/LYPD1 axis promotes the development of liver cancer [104]. HBXIP has been found to be upregulated in liver cancer tissues and to be associated with poor prognosis. HBXIP can modify hypoxia-inducible factor-1α (HIF-1α) through m6A, mediated by METTL3, and drive metabolic reprogramming in HCC cells [105]. Xu et al. suggested that SUMOylated METTL3 promotes the progression of HCC and may act through the UBC9/SUMOylated METTL3/Snail axis [106]. METTL3 promotes m6A modification of pri-miR-873-5p and helps increase the expression of miR-873-5p. METTL3 also promotes tumour formation in nude mice by downregulating the expression of SMG1. METTL3 inhibits the expression of SMG1 through upregulation of miR-873-5p expression, mediated by m6A modification, thereby playing a role in promoting HCC development [107]. METTL3 expression is significantly downregulated in human sorafenib-resistant HCC [108]. The absence of METTL3 under hypoxic conditions promotes the resistance of cultured liver cancer cells to sorafenib, enhances and the expression of angiogenic genes and activates autophagy-related pathways. FOXO3 is a key downstream target of METTL3, and m6A modification of the 3´ untranslated region of FOXO3 mRNA improves its stability through a YTHDF1-dependent mechanism. Analysis of clinical samples further showed that the levels of METTL3 and FOXO3 expression are closely related in patients with liver cancer are closely related. In a mouse xenograft model, METTL3 deletion significantly enhanced the resistance of liver cancer to sorafenib by disrupting the METTL3-mediated stability of FOXO3 mRNA, while FOXO3 overexpression restored the sensitivity of liver tumours to sorafenib. FOXO3 is an important target of m6A modification in the development of therapeutic resistance to sorafenib therapy in liver cancer [108]. FTO plays a protective role in the development of HCC in the body, especially in the initial stage [109]. The deacetylase SIRT1 is a key regulator of FTO expression downregulation, and it also plays a role through SUMO modification. SIRT1 downregulates FTO expression through RANBP2-mediated SUMOylation and influences m6A RNA modification of tumour suppressors such as GNAO1 that mediate the development of liver cancer, thereby exerting oncogenic effects [110]. Some researchers have found that the overexpression of FTO is related to poor prognosis in patients with liver cancer. FTO gene knockout inhibits tumour growth and proliferation in vivo and induces G0/G1 cell cycle arrest [111]. These two opposing views are worthy of further exploration. Zuo et al. found that m6A modification is the cause of upregulated LINC00958 expression in liver cancer. LINC00958 was found to promote HCC cell proliferation, migration, and invasion and adipogenesis through the miR-3619-5p/HDGF axis. In addition, Zou et al. used a PDX model to confirm the effect of LINC00958 on the growth of HCC in vivo. They also developed a PLGA-based nanoplatform for encapsulating LINC00958 siRNA and determined that this drug release system has the characteristics of controlled release, targeting, safety, and good antitumour effects [112]. Mutation and copy number alteration of m6A regulatory factors lead to a decline in cell survival, which is closely related to TP53 mutation in HCC [113]. Fusaric acid (FA) epigenetically reduces the expression of p53 by its altering promoter methylation and m6A RNA methylation in HepG2 cells [114]. Zhou et al. suggested that METTL3 and YTHDF1 are independent factors affecting the prognosis of patients with HCC. Patients with different co-expression levels of METTL3 and YTHDF1 were found to have different survival outcomes. The combination of METTL3 and YTHDF1 expression can be used as a biomarker reflecting the degree of malignancy to evaluate prognosis in HCC [115]. METTL3 is a biomarker of poor prognosis, and the abnormal upregulation of METTL3 expression in HCC results from CNV and DNA methylation rather than somatic mutation [116]. The low-expression of demethylases (mainly FTO and ALKBH5) and concomitant m6A modifications in noncancerous peritumoral liver tissues are believed to enhance the malignant potential of liver cancer after resection [117]. Overall, the main m6A regulatory factors related to HCC are YTHDF2, YTHDF1, METTL3, KIAA1429 and ZC3H13. Scholars later proposed prognostic marker signatures composed of these five components to identify patients with high-risk cancer [118–120]. In patients with high-risk cancer, ZC3H13 expression is upregulated, while the expression of METTL3, KIAA1429, YTHDF1 and YTHDF2 is downregulated [120]. More importantly, univariate and multivariate Cox regression analyses showed that the signature-based risk score is an independent prognostic factor in patients with HCC. These five m6A RNA methylation regulators can be used as practical and reliable prognostic tools for HCC and may have potential value as therapeutic targets [119]. Zhang et al. developed a deep learning framework, DeepM6ASeq, to predict sequences containing m6A and to describe their surrounding biological characteristics based on miCLIP-seq data, which detects m6A sites at a single-base resolution. Compared with other learning classifiers, DeepM6ASeq showed better performance. The motifs identified using DeepM6ASeq corresponded to the known m6A readers. In addition, DeepM6ASeq identified a new m6A reader, FMR1 [121].

#### Pancreatic ductal adenocarcinoma (PDAC)

The level of RNA m6A modification in PDAC tissue has been confirmed to be significantly higher than that in adjacent tissues [122, 123]. METTL3 increases m6A modification in pancreatic cells, and METTL3 can promote the proliferation and invasion of pancreatic cancer cells [122]. Taketo et al. also found that pancreatic cancer cells lacking mettl3 showed higher sensitivity to anticancer agents such as gemcitabine, 5-fluorouracil, and cisplatin, as well as to irradiation, but the morphology and proliferation of these cellswere not affected. It is speculated that METTL3 may regulate the MAPK cascade and cellular processes, leading to resistance to chemotherapy and radiotherapy in pancreatic cancer cells [122]. Xia et al. found that miR-25-3p expression is significantly higher in smokers than non-smokers and obviously higher in PDAC than in non-tumour tissues and that an increased level of miR-25-3p is associated with a shortened patient survival time. Smoke condensate induces hypomethylation of the METTL3 promoter, leading to METTL3 overexpression, which subsequently increases the m6A modification of pri-miR-25. The oncogenic effects of abnormal miR-25-3p expression include inhibition of PHLPP2 and activation of AKT-p70S6K oncogenic signaling, forming the METTL3-miR-25-3p-PHLPP2-AKT axis, as well as the promotion of PDAC development in people who smoke [124]. In addition, Xie et al. discovered that the nuclear protein NKAP might be a reader of m6A on pri-miR-25 [124]. METTL14 is also the main enzyme regulating the frequency and location of m6A methylation. The increase in the m6A methylation levels in pancreatic cancer is caused by an imbalance in the level of the m6A regulatory factor METTL14. Upregulation of METTL14 expression can demonstrably promote the proliferation and migration of pancreatic cancer cells in an m6A-dependent manner by directly targeting the mRNA of the downstream effector PERP (the p53 effector associated with PMP-22) [123]. Targeted adenosine methylation in pancreatic cancer leads to increased PERP mRNA turnover, thereby reducing PERP (mRNA and protein) levels [123]. YTHDF2 has the dual function of promoting and inhibiting the progression of pancreatic tumours [125]. YTHDF2 coordinates two cellular processes. One function of YTHDF2 is to coordinate the “migration and proliferation dichotomy,“ involving the promotion of the proliferation of pancreatic cancer cells and simultaneous inhibition of their migration and invasion; the other is to regulate EMT of pancreatic cancer cells. YTHDF2 expression is upregulated at both the mRNA and protein levels in pancreatic cancer in humans and serves as an independent factor predicting a high stage in patients. YTHDF2 may be an indicator of the diagnosis and prognosis of pancreatic cancer, and YTHDF2 has potential value as a new target for the prevention and treatment of pancreatic cancer [125]. Deletion of ALKBH5 is related to poor clinicopathological characteristics and prognosis in pancreatic cancer (PC). ALKBH5 overexpression reduces PC cell proliferation, migration, and invasion and tumour growth, while ALKBH5 deletion promotes the progression of PC [126]. ALKBH5 inhibits the progression of pancreatic cancer through the m6A-YTHDF2-dependent post-transcriptional activation of PER1 [126]. In addition, ALKBH5 can demethylate KCNK15-AS1 and is negatively correlated with m6A modification. The expression of KCNK15-AS1 lncRNA has been found to be decreased in pancreatic cancer tissue, thus inhibiting the metastasis of pancreatic cancer cells [127]. ALKBH5 overexpression can render PDAC cells sensitive to gemcitabine and inhibit PDAC tumorigenesis by reducing the m6A modification level of WIF-1 and blocking the activation of Wnt signaling [128]. m6A demethylation is an important methylation removal mechanism that regulates mRNA stability in pancreatic cancer cells. FTO is overexpressed in pancreatic cancer cells. FTO regulates the proliferation of pancreatic cancer cells by regulating cell cycle progression. FTO knockdown affects the stability of c-Myc mRNA, thereby inhibiting DNA synthesis [129]. Upregulation of IGF2BP2 expression is associated with poor prognosis in patients with pancreatic cancer, and inhibiting IGF2BP2 impedes cell proliferation. IGF2BP2 works by regulating the lncRNA DANCR. IGF2BP2 works synergistically with DANCR to regulate its stability. In normal cells, the level of IGF2BP2 is low; thus, its ability to interact with and stabilize DANCR is limited. In tumour cells, IGF2BP2 expression is upregulated, which increases the opportunity for IGF2PB2 to interact with DANCR and stabilize it, especially when the RNA methylation mechanism is dysregulated. As a result, these tumour cells become more proliferative and more resistant to drugs [130]. IGF2BP2 has also been found through TCGA database analysis and various bioinformatics methods to play an important role in m6A modification in PAAD from genomics, transcriptomics, and clinical data perspectives [131]. The m6A regulatory genes are differentially expressed in PAAD and are closely related to the clinicopathological characteristics of PAAD [132]. IGF2BP2, KIAA1429 and HNRNPC have been found to be related to various biological behavior, such as the adipocytokine signaling pathway, benign/poorly differentiated tumour pathway, tumour metastasis pathway, epithelial-mesenchymal transition pathway, gemcitabine resistance pathway, and stemness pathway [133]. Some researchers have proposed that, as in liver cancer, new risk signatures constructed from 5 m6A-related genes (METTL3, METTL14, KIAA1429, ALKBH5 and YTHDF1) can be used as independent prognostic factors for PAAD [134]. Some researchers have also identified differential genes related to PAAD survival. These genes have abundant m6A modification sites. These sites are potential targets of m6A-related genes involved in pancreatic tumorigenesis and in the malignant progression of PC [135, 136]. There is also a potential correlation between m6A-related genes and the immune microenvironment of pancreatic cancer, indicating that the composition of infiltrating immune cells may affect m6A modification of tumour cell genes [132, 135].

#### m6A modulators as therapeutic targets in digestive system tumours

m6A modification is regulated by methyltransferases, demethylases and RNA-binding proteins. Changes in the abovementioned complexes will cause changes in expression levels, leading to the occurrence, development and invasion of tumours. Therefore, modulators or inhibitors of m6A modification may be potential agents for the treatment of malignant tumours. In terms of chemoradiotherapy, overexpression of the demethylase ALKBH5 can increase the sensitivity of PC cells to chemotherapy [128]. PC cell lines with knockout of METTL3 are highly sensitive to antitumour drugs such as gemcitabine, 5-fluorouracil, and cisplatin, as well as to radiotherapy, suggesting that METTL3 is an effective target for improving the efficacy of PC therapies [137]. In EC, the expression of METTL3 is related to recurrence, suggesting that METTL3 overexpression may be a key factor in the resistance to platinum drugs in patients with esophageal cancer. Therefore, regulating the expression of METTL3 may be a new method to improve the efficacy of platinum drugs [138]. In terms of immunotherapy, knockout of FTO gene expression can increase the sensitivity of tumour cells to immunotherapy [139]. Li et al. found that m6A modification regulates T cell homeostasis by targeting the IL-7/STAT5/SOCS pathway. m6A modification enzymes, as key regulators of T cells, play an important role in the regulation of the immune system and tumour growth and the spread of tumours [140]. In terms of targeted therapy, Huang et al. discovered a highly selective FTO inhibitor, meclofenamic acid (MA), which can compete with FTO for its binding site and increase m6A modification to inhibit tumour progression [141].In addition, carbonic anhydrase IV (CA4) can inhibit the Wnt pathway by targeting WTAP, thereby inhibiting tumour cell proliferation and inducing cell cycle arrest in colon cancer [142].

## Discussion

m6A modification has attracted increasing attention with the development of high-throughput sequencing and the popularization of databases such as TCGA, and researchers have discovered that m6A modifications are ubiquitous in humans and inseparable from the occurrence and development of tumours. Several scholars have conducted research on m6A modifications, and an increasing number of regulatory factors, such as KIAA1429 and ZC3H13, have been discovered. It has also become clear that m6A modification is a complex and multi-mechanism process and that regulatory factors may play a dual role in tumorigenesis and development. For example, YTHDF2 can directly bind to the m6A-modified site in the EGFR 3’-UTR to promote the degradation of EGFR mRNA in HCC cells, thereby inhibiting HCC growth and proliferation [95]. On the other hand, YTHDF2 can promote tumour metastasis [96]. This observation suggests that it may be possible to fully activate the tumour suppressor pathway by inhibiting the cancer-promoting pathway mediated bycertain regulatory factors to improve patient survival.

In the last few years, research on m6A in cancer has mainly focused on its role in lung cancer, glioma and blood system diseases. It has been found in recent years that m6A modification is also common in cancers of the digestive tract, as discussed in this review. Relevant research results can be translated to the clinic more quickly due to the wide application and popularization of databases such as TCGA. Most studies have shown that the expression of m6A regulators can be used as a marker for tumour growth, progression, and prognosis. Some scholars have summarized a standard m6A score, compared it with the combined positive score (CPS), and speculated that this standard m6A score might be able to guide the development of treatment plans including immunotherapy [61]. This finding undoubtedly proves that tumour formation is a complex pathogenic process with multiple mechanisms that make it difficult to find a cure and can result in recurrence after complete remission.

There are some limitations of this review since m6A in digestive system tumours has only recently and gradually attracted attention. For example, there is little research on EC, and most studies have focused on ESCC. Most scholars have mainly focused on popular regulators such as METTL3, YDH family members, ALKBH5 and FTO. Many scholars are engaged in further exploration of the mechanism underlying m6A modifications. The regulation of m6A-related genes may be a new cancer treatment strategy that is worthy of further exploration. The specific mechanisms by which m6A modification regulates different pathways and the specificity and sensitivity of m6A as a tumour marker need to be further studied. Finally, whether there are more correlations between m6A modification and the sensitivity, drug resistance and long-term prognosis of patients with digestive system tumours remains to be determined. In-depth research on m6A modification in digestive system tumours may provide new directions for clinical prediction and further treatment.

Summary and prospect In recent years, with the development of m6A modification detection technology, research on the role of m6A modification and its related enzymes in tumors has made substantial progress. m6A modification is a “double-edged sword”. Hypermodification of some genes may change the splicing and translation capability of the corresponding RNA, leading to the occurrence and development of malignant tumors, while lack of m6A modification on some genes may lead to tumorigenesis. Due to the heterogeneity of tumors, the same writer complex, eraser complex, and reader complex have different effects in different tumors; these complexes can act as oncogenes or tumor suppressor genes, but all are triggered or promoted by the dysregulation of protein expression. This observation indicates the direction for tumor treatment. Although many regulatory mechanisms have been discovered, there are still many unclear mechanisms and controversies due to the complexity of m6A regulation and other factors. Future research on m6A modification capabilities will be a major breakthrough, in which the desired level of m6A methylation for recovery is the key to treatment. The discovery of more m6A modification enzyme modulators and competitive antagonists is important for the development of accurate and effective m6A-targeted drugs.

## Data Availability

The data supporting the conclusion of this review have been included within the article.
